# A Density Functional Theory and Information-Theoretic Approach Study of Interaction Energy and Polarizability for Base Pairs and Peptides

**DOI:** 10.3390/ph15080938

**Published:** 2022-07-28

**Authors:** Dongbo Zhao, Shubin Liu, Dahua Chen

**Affiliations:** 1 Institute of Biomedical Research, Yunnan University, Kunming 650500, China; dongbo@ynu.edu.cn; 2 Research Computing Center, University of North Carolina, Chapel Hill, NC 27599-3420, USA; 3 Department of Chemistry, University of Nortrefh Carolina, Chapel Hill, NC 27599-3290, USA

**Keywords:** density functional theory (DFT), information-theoretic approach (ITA), base pairs, peptides, molecular polarizability

## Abstract

Using density functional theory (DFT) and the information-theoretic approach (ITA) quantities to appreciate the energetics and properties of biopolymers is still an unaccomplished and ongoing task. To this end, we studied the building blocks of nucleic acid base pairs and small peptides. For base pairs, we have dissected the relative importance of energetic components by using two energy partition schemes in DFT. Our results convincingly show that the exchange-correlation effect predominantly governs the molecular stability of base pairs while the electrostatic potential plays a minor but indispensable role, and the steric effect is trivial. Furthermore, we have revealed that simple density-based ITA functions are in good relationships with molecular polarizabilities for a series of 30 hydrogen-bonded base pairs and all 20 natural *α*-amino acids, 400 dipeptides, and 8000 tripeptides. Based on these lines, one can easily predict the molecular polarizabilities of larger peptides, even proteins as long as the total molecular wavefunction is available, rather than solving the computationally demanding coupled-perturbed Hartree–Fock (CPHF) equation or its DFT counterpart coupled-perturbed Kohn–Sham (CPKS) equation.

## 1. Introduction

Nucleic acids and proteins are important biological polymers in living organisms. Proteins are responsible for the catalysis of biological processes, while nucleic acids or base pairs play a role as carriers of the genetic information. However, *ab initio* calculations of entire nucleic acids or proteins are still a tough nut to crack or even computationally intractable. Rather, one can easily delve into their building blocks, base pairs of nucleic acids and amino acids of proteins or peptides, respectively.

For base pairs, there exists a type of important noncovalent interaction: hydrogen-bonding interactions, which could stabilize the two strands of nucleic acids (DNA and RNA). To figure out the origin and nature of hydrogen-bonding interactions in base pairs is still of heated discussion and an ongoing task in the literature [1,2,3,4,5]. Normally, the role of electrostatic potential is greatly emphasized [2], and in most cases, this is the dominantly important energetic component of the interaction energy. However, this is not the whole picture of H-bonded interactions! Parthasarathi et al. [4] have shown that the presence of critical points between H-bonded DNA base pairs is indicative of the closed-shell kind of interactions. With Bader’s quantum theory of atoms in molecules (QTAIM), Toczyłowski et al. [5] have revealed that the electrostatic and exchange energies are found to be the most important components of the overall interaction energy, although the dispersion and the induction energies also play important roles. Of note, the exchange term used is not the same quantity as that in DFT [6]. In this work, we also resort to the supermolecule interaction model as that of Toczyłowski et al. We employed the energetic components in DFT to dictate which is responsible for the molecular stability. From two energy decomposition schemes [6,7], we have clearly shown that the exchange-correlation effect in DFT predominantly governs the molecular stability of base pairs while the electrostatic potential plays a minor but indispensable role, and the steric effect is trivial. 

Besides energetics, we have also explored the molecular polarizabilities of 30 base pairs and 20 natural *α*-amino acids, 400 dipeptides and 8000 tripeptides. A few simple density-based ITA functions, such as Shannon entropy [8], Fisher information [9], Ghosh−Berkowitz−Parr entropy [10], Onicescu information energy [11], relative Rényi entropy [11], information gain [12], and relative Fisher information [13,14] are utilized to correlate with molecular polarizabilities. In addition, molecular volumes are also employed. We have found that there exist strong linear correlations between molecular polarizabilities and ITA quantities. The implication of these lines is straightforward, that one can easily predict the molecular polarizabilities of larger peptides, or even an entire protein when the molecular wavefunction can be obtained. In other words, one can bypass the time-consuming CPHF/CPKS equation [15,16,17] by numerically integrating some density-based functions. Moreover, our work on molecular polarizability is physics-based (locality of electron density) and is markedly different from other mathematics-based predictions, such as machine learning (ML) [18,19,20] and regression model [21].

## 2. Results

### 2.1. Validation

Hait et al. [22] have verified that hybrid density functionals are satisfactory in calculating molecular polarizabilities. A few popular density functionals, such as M06-2X [23], B3LYP [24,25], CAM-B3LYP [26], PBE0 [27] and ωB97XD [28] were chosen together with a series of atomic-centered Gaussian-type basis sets 6-311G(d,p) [29], Def2-SVP [30], Def2-TZVP [30] and aug-cc-pVTZ [31]. We calculated the molecular polarizabilities for a total of 20 natural α-amino acids (in its neutral form in the gas phase) at different theoretical levels as shown in Table 1. The MP2 results from the literature are also collected for comparison. Two statistical parameters, mean unsigned error (**MUE**) and mean signed error (**MSE**), are employed to gauge the quality of the method. The experimental values [32] of *α*_iso_ are taken as reference to obtain **MUE** and **MSE** values. It is clearly shown that the combination of 6-311G(d,p) with 5 functionals from this work or MP2 (**MSE** = ~16 Bohr^3^) from the literature [33] leads to large deviations compared with the experiment. This is also the case for a small double-zeta basis set Def2-SVP, when combined with M06-2X, B3LYP, PBE0, and ωB97XD, with an exception of CAM-B3LYP. The reason behind is unknown. When it comes to a relatively larger triple-zeta basis set Def2-TZVP, the **MSE** dramatically decreases, for example from 18.4 (Def2-SVP) to 8.9 Bohr^3^ for M06-2X. If an even better basis set aug-cc-pVTZ is used, the **MSE** is only 3.8 Bohr^3^. Of note, the relative deviation of 5 density functionals should be comparable when combined with, for example, Def2-TZVP. In addition, though B3LYP performs well in predicting molecular polarizabilities here, its failure in delineating dispersion will undoubtedly hinder the wide applicability of ITA quantities for various kinds of inorganic, organic and biological systems. The results will be presented in our forthcoming publication. Collectively, with both accuracy and efficiency taken into consideration, we selected M06-2X/Def2-TZVP for predicting molecular polarizabilities.

### 2.2. Total Energy Decomposition of Base Pairs

**Figure 1:**
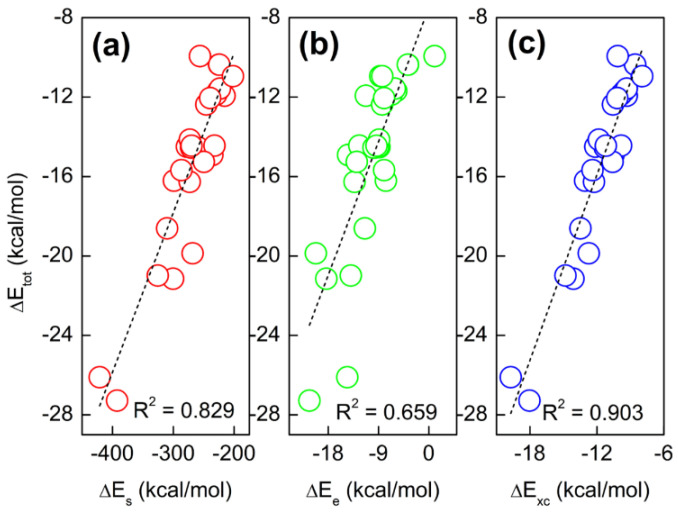
Strong correlations between the total energy difference (ΔE_tot_) and (**a**) steric hindrance (ΔE_s_), (**b**) electrostatic potential (ΔE_e_), and (**c**) exchange-correlation effect (ΔE_xc_). The *y*-axis of (**a**) spans to (**b**,**c**).

**Figure 2:**
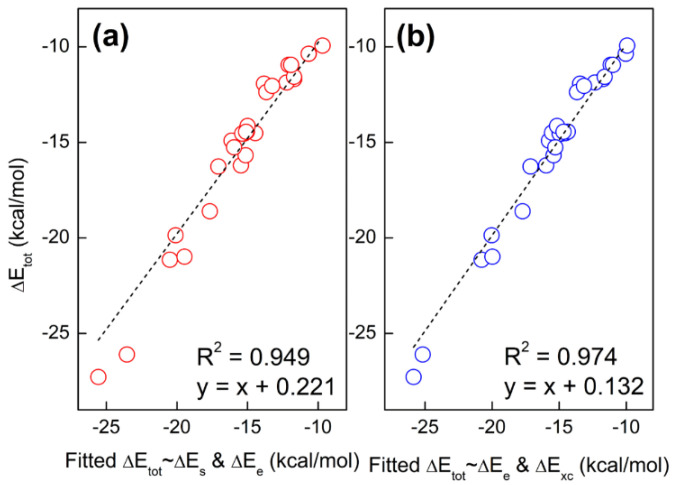
Two-variable fit of the total energy difference (ΔE_tot_) with (**a**) steric hindrance (ΔE_s_) and electrostatic potential (ΔE_e_) and (**b**) electrostatic potential (ΔE_s_) and exchange-correlation effect (ΔE_xc_).

To determine the nature of hydrogen bonding interactions in base pairs is of heated discussion in the literature. In this work, we aim to use two energy decomposition schemes as mentioned above to dictate which energetic component is responsible for the hydrogen bonding interactions. Using the supramolecular approach, the interaction energy (ΔE) and its components, including kinetic (ΔT_s_), exchange-correlation (ΔE_xc_), electrostatic potential (ΔE_e_), steric hindrance (ΔE_s_), and quantum effect (ΔE_q_), are obtained at the M06-2X/Def2-TZVP level, as collected in Table 2. Here, we only consider the electronic energy, with thermal contributions and BSSE (basis set superposition error) [34,35] corrections neglected. The total energy difference is always negative, indicating that hydrogen-bonding interactions are energetically favorable. In addition, one can see that exchange-correlation (ΔE_xc_), electrostatic potential (ΔE_e_), and steric hindrance (ΔE_s_) all make positive contributions to the total energy difference. However, the large negative values of steric hindrance are compensated by the positive Fermionic quantum effect in the new scheme. In the conventional scheme, the positive kinetic energy also cancels out part of the exchange-correlation effect and electrostatic potential. Next, we will figure out the relative importance of these three components. Shown in Figure 1 are the linear correlations between the total energy difference (ΔE) and ΔE_s_, ΔE_e_, and ΔE_xc_. These lines indicate that single-variable linear regression cannot distinguish the relative importance of these three energetic components. To resolve this issue, two-variable fits are used to make that happen. Shown in Figure 2 are the strong linear correlations between the fitted and predicted total energy difference. The fitted regression equations for Figure 2a,b are

ΔE = 0.040ΔE_s_ + 0.467ΔE_e_

and

ΔE = 1.017ΔE_xc_+ 0.350ΔE_e_

It is lucidly shown that it is the exchange-correlation effect that dominates the hydrogen-bonding interactions, while the electrostatic potential also plays an important role and the steric hindrance is trivial.

### 2.3. Molecular Polarizabilities of Base Pairs

In Table 3, we have collected the molecular polarizabilities, molecular volumes, and ITA quantities for 30 base pairs, which are obtained at the M06-2X/Def2-TZVP level. Also included in Table 3 are the correlation coefficients (R^2^) between the molecular polarizabilities and molecular volumes and ITA quantities. One can observe that Shannon entropy (S_S_), Fisher information (I_F_), 2nd and 3rd relative Reényi entropy (^r^R_2_ and ^r^R_3_), and G_3_ are in strong linear relationships with molecular polarizabilities, with R^2^ > 0.8. It is intriguing to note that the absolute values of G_3_ are very close to molecular polarizabilities. However, the reason is unknown at present. In Table 4, we compare the polarizability results from conventional calculations, G_3_ data, and those predicted on top of the molecular wavefunctions obtained at the M06-2X/Def2-TZVP level. One can easily observe that the G_3_ data are very accurate in comparison to conventional polarizability data with MSE(%) to 1.5. However, the two sets of predicted data employing the original Tkatchenko–Scheffler (TS) formula [36] on top of Becke [37] or Hirshfeld [38] partitions are either strongly underestimated or overestimated, with MUE(%) up to –23.4 and 11.6, respectively. It is found that a mean value can greatly reduce the MSE(%) to 5.9. Though a rational theoretical explanation is lacking at the moment, we can take a careful look of the TS formula, $\alpha_{\mathrm{mol}}^{\mathrm{TS}-\mathrm{old}}=\underset{A}{\sum }\alpha_{A}^{\mathrm{eff}}=\underset{A}{\sum }\alpha_{A}^{\mathrm{free}}\left(V_{A}^{\mathrm{eff}}/V_{A}^{\mathrm{free}}\right),$ where the sum runs over all atoms in a molecule. The weights $\;\left(V_{A}^{\mathrm{eff}}/V_{A}^{\mathrm{free}}\right),\;$measuring the volume ratio for atom A in a molecule to the free atom A in vacuum, can be obtained by the Becke or Hirshfeld partitioning of the electron density. Of note, $\alpha_{A}^{\mathrm{free}}$ denotes the atomic polarizabilities and they have been obtained accurately [39]. Thus, the main problem should come from the weights. Very recently, a revised formula $\alpha_{\mathrm{mol}}^{\mathrm{TS}-\mathrm{new}}=\underset{A}{\sum }\alpha_{A}^{\mathrm{eff}}=\underset{A}{\sum }\alpha_{A}^{\mathrm{free}}{\left(V_{A}^{\mathrm{eff}}/V_{A}^{\mathrm{free}}\right)}^{4/3}$ has been proposed for small molecules with improved performance [40]. Yet, the performance of both original TS method and its variant for macromolecules requires more extensive work. 

### 2.4. Molecular Polarizabilities of Amino Acids, Dipeptides and Tripeptides

**Figure 3:**
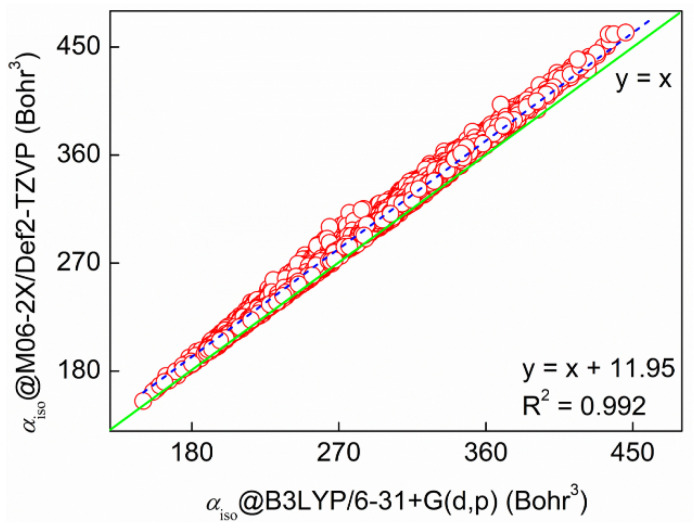
Cross-validation of molecular polarizabilities of 8000 tripeptides carried out at the M06-2X/Def2-TZVP//M06-2X/Def2-SVP and B3LYP/6-31+G(d,p)//B3LYP/6-31G level, respectively.

**Figure 4:**
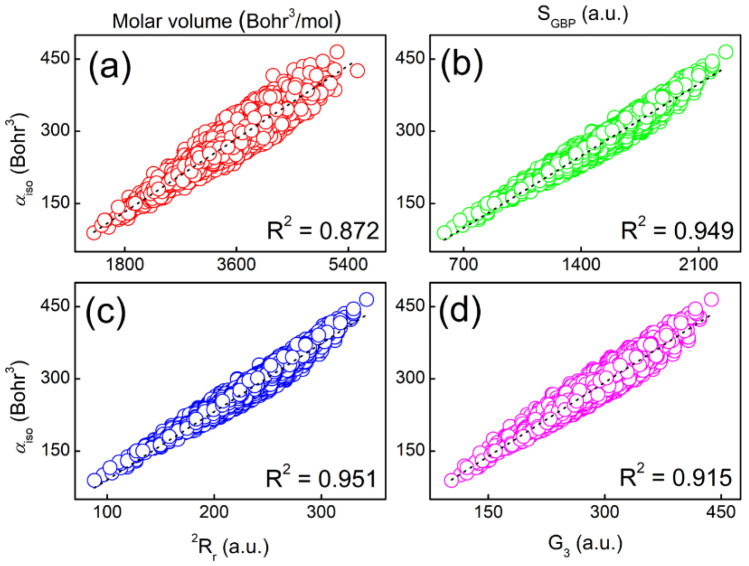
Strong correlations between molecular polarizability and (**a**) molecular volume, (**b**) S_GBP_, (**c**) ^2^R_r_, and (**d**) G_3_ for a total of 20 amino acids, 400 dipeptides, and 8000 tripeptides. Computations were carried out at the M06-2X/Def2-TZVP//M06-2X/Def2-SVP level.

We first cross-validate the accuracy of the molecular polarizabilities of 8000 tripeptides. The two theoretical levels are M06-2X/Def2-TZVP//M06-2X/Def2-SVP and B3LYP/6-31+G(d,p)//B3LYP/6-31G level, respectively. Shown in Figure 3 is the strong correlation coefficient (R^2^ = 0.992) between the two sets of calculated molecular polarizabilities. The gap between the two methods is only 11.95 Bohr^3^, indicating that one can use a relatively cheaper theoretical method to obtain accurate results. In addition, we have found some strong correlations for a total of 20 amino acids, 400 dipeptides, and 8000 tripeptides between molecular polarizabilities and molecular volumes, GBP entropy (S_GBP_), 2nd relative Rényi entropy (^r^R_2_), and G_3_ as shown in Figure 4. The correlation coefficients are in the range of 0.87 to 0.95. We have to point out that molecular polarizabilities can be linearly correlated with molecular volumes at both atomic and molecular levels [41,42,43,44,45,46,47,48]. Here, we have verified that ITA quantities can serve as good indicators of molecular properties, in our case, molecular polarizability. This means that one can directly predict molecular polarizabilities of larger peptides, or even proteins. It is well-documented that it is time-consuming to solve the CPHF/CPKS equations [15,16,17]. When the molecular system becomes larger, it may be even intractable. For ITA quantities, as long as the total molecular wavefunction is obtained in a single-point calculation, numerical integration of ITA quantities normally requires much less time than iteratively solving the CPHF/CPKS equations. It is anticipated when it comes to real systems, like proteins, one can combine the linear-scaling fragment-based methods, such as GEBF (generalized-energy based fragmentation) [49,50,51,52], and ITA quantities to accurately and efficiently predict the molecular polarizabilities.

## 3. Discussion

We have systematically investigated the energetics of 30 base pairs and molecular polarizabilities of 30 base pairs and 20 amino acids, 400 dipeptides, and 8000 tripeptides. It is the first time that ITA quantities have been applied to correlate with molecular polarizabilities. Though at present, in theory we have not verified the existence of such a linear correlation between ITA quantities and molecular polarizabilities, they are both related to electron density and its derivatives. Additionally, our results presented here demonstrate that one can determine the molecular polarizability by numerically integrating the simple density-based functions when the molecular wavefunction of a given system is obtained from single-point calculations, rather than resorting to iteratively obtaining the molecular orbital derivatives. The implication of our work is straightforward, that one can use the linear relationships as shown in the text to predict molecular polarizabilities of relatively larger peptides, or even larger proteins. Can the ITA quantities be widely applied to various inorganic, organic and biological systems? More work along this line is required.

We have to point out that we are not the first to predict the molecular polarizabilities just as employing the electron density as the input. Jayatilaka et al. [53] has cast the molecular polarizability in terms of moments of the ground-state electron density matrix and the results are reasonably good against CPHF results. The corresponding performance for hyperpolarizability is far from satisfactory. Since hyperpolarizability is not considered in this work, we will figure out if the abovementioned density-based ITA functions can predict the molecular hyperpolarizability well. 

## 4. Materials and Methods

### 4.1. Energy Decomposition Schemes in DFT

In Kohn–Shan DFT, the total energy difference (Δ*E*) can be decomposed into its components via [3,4]

(1) $$\Delta E\left[\rho \right]=\Delta T_{s}\left[\rho \right]+\Delta E_{e}\left[\rho \right]+\Delta E_{xc}\left[\rho \right]\;$$

and

(2) $$\Delta E\left[\rho \right]=\Delta E_{s}\left[\rho \right]+\Delta E_{e}\left[\rho \right]+\Delta E_{q}\left[\rho \right]\;\;$$

where *T*_s_, *E*_e_, and *E_xc_* are the noninteracting kinetic, electrostatic, and exchange-correlation terms, respectively. The electrostatic potential *E_e_* has three components: the nuclear−electron attraction, (*V*_ne_), the classical Coulombic repulsion, (*J*), and the nuclear−nuclear repulsion (*V*_nn_). The last term, *E*_xc_, consists of exchange (*E_x_*) and correlation (*E*_c_) components. In Equation (2), *E*_s_ stands for the steric hindrance, and *E*_q_ signifies the contribution from Fermionic quantum effect. The steric effect *E*_s_ has been proved to be simply the Weizsäcker kinetic energy,

(3) $$\tau_{W}\left(r\right)=\frac{1}{8}\frac{{\left|\nabla \rho \left(r\right)\right|}^{2}}{\rho \left(r\right)}\;\;$$

Combining Equations (1)–(3), one can simply define *E_q_*, which reads

(4) $$\;\Delta E_{q}\left[\rho \right]=\Delta E_{xc}\left[\rho \right]+\Delta T_{s}\left[\rho \right]-\Delta E_{s}\left[\rho \right]\;\;$$

This new formulation has its own distinct physical meaning with a corresponding physical state. It has been applied to a number of molecular systems and phenomena, whose results are consistent with our chemical intuition and conventional wisdom [54].

### 4.2. Information-Theoretic Approach Quantities

Shannon entropy *S*_S_ [8] is a measure of the spatial delocalization of the electron density, and Fisher information *I*_F_ [9] measures the sharpness or localization of the same. They are defined as Equations (5) and (6), respectively.

(5) $$S_{S}=-\int^{\;}\rho \left(r\right)\ln\rho \left(r\right)dr$$

(6) $$\;I_{F}=-\int^{\;}\frac{{\left|\nabla \rho \left(r\right)\right|}^{2}}{\rho \left(r\right)}dr\;$$

Additionally, for atoms and molecules, *I*_F_ has an equivalent expression [55] in terms of the Laplacian of the electron density, ∇^2^*ρ*(**r**).

(7) $$I_{F}^{'}=-\int^{\;}\left[\nabla^{2}\rho \left(r\right)\right]\ln\rho \left(r\right)dr\;$$

Ghosh−Berkowitz−Parr (GBP) entropy *S*_GBP_ [10]

(8) $$S_{\mathrm{GBP}}=-\int^{\;}\frac{3}{2}k\rho \left(r\right)\left[c+\ln\frac{t\left(r;\rho \right)}{t_{\mathrm{TF}}\left(r;\rho \right)}\right]dr$$

where *t*(**r**; *ρ*) is the kinetic energy density, which is related to the total kinetic energy *T*_S_ via

(9) $$\int^{\;}t\left(r;\rho \right)dr=T_{S\;}$$

*t*_TF_(**r**; *ρ*) is the Thomas−Fermi (TF) kinetic energy density given by

(10) $$t_{\mathrm{TF}}\left(r;\rho \right)=c_{K}\rho^{5/3}\left(r\right)\;$$

with *K* as the Boltzmann constant (set to be unity for convenience in this work), *c* = (5/3) + ln(4*πc_K_*/3), and *c*_K_ = (3/10)(3*π*^2^)^2/3^, the specific form of the local kinetic energy

(11) $$t\left(r;\rho \right)=\underset{i}{\sum }\frac{1}{8}\frac{\nabla \rho_{i}\Delta \nabla \rho_{i}}{\rho_{i}}-\frac{1}{8}\nabla^{2}\rho$$

More recently, additional ITA quantities have been introduced as new reactivity descriptors in conceptual density functional theory (CDFT) [11]. One example is Onicescu information energy [11] of order *n*

(12) $$E_{n}=\frac{1}{n-1}\int^{\;}\rho^{n}\left(r\right)dr\;$$

relative Rényi entropy [8] of order *n*

(13) $$R_{n}^{r}=\frac{1}{n-1}\ln\left[\int^{\;}\frac{\rho^{n}\left(r\right)}{\rho_{0}^{n-1}\left(r\right)}dr\right]\;\;$$

and information gain [12] (also called Kullback−Leibler divergence) *I*_G_ is given in Equation (14)

(14) $$I_{G}=\int^{\;}\rho \left(r\right)\ln\frac{\rho \left(r\right)}{\rho_{0}\left(r\right)}dr\;$$

where *ρ*_0_(**r**) is the reference-state density satisfying the same normalization condition as *ρ*(**r**). 

Very recently [13,14], we have proposed another three functions *G*_1_, *G*_2_, and *G*_3_, whose analytical forms as shown below:

(15) $$G_{3}=\underset{A}{\sum }\int^{\;}\rho_{A}\left(r\right){\left[\nabla \ln\frac{\rho_{A}\left(r\right)}{\rho_{A}^{0}\left(r\right)}\right]}^{2}dr\;$$

(16) $$G_{1}=\underset{A}{\sum }\int^{\;}\nabla^{2}\rho_{A}\left(r\right)\frac{\rho_{A}\left(r\right)}{\rho_{A}^{0}\left(r\right)}dr\;\;$$

(17) $$G_{2}=\underset{A}{\sum }\int^{\;}\rho_{A}\left(r\right)\left[\frac{\nabla^{2}\rho_{A}\left(r\right)}{\rho_{A}\left(r\right)}-\frac{\nabla^{2}\rho_{A}^{0}\left(r\right)}{\rho_{A}^{0}\left(r\right)}\right]dr\;\;$$

The *G*_2_ function involves Laplacian contribution and can be regarded as the relative Laplacian contribution to the steric potential. The quantifications and applications of Equations (15)–(17) can be found in [14]. Suffice to note, during the past decade, we have attempted to seamlessly glue the density functional theory and information theory together, as electron density can be used as a linker of these two theories. The progress and applications can be found in our recent review [54]. For example, very recently we have applied the information-theoretic approach to appreciate homochirality [56,57], which is another very fundamental problem in biology and we will look into it in the near future.

### 4.3. Computational Details

#### 4.3.1. Base Pairs

**Scheme 1:**
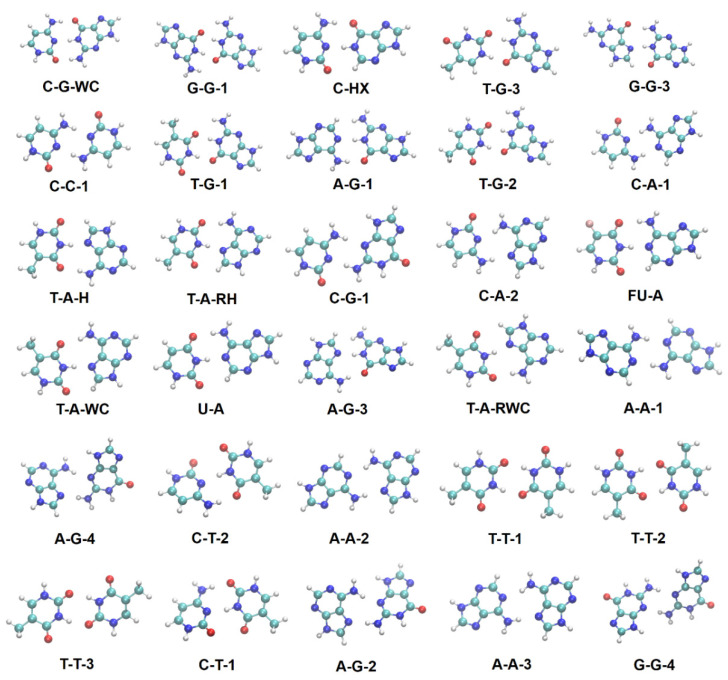
Optimized structures of the 30 base pairs. Color code: H in white, C in cyan, N in blue, O in red, and F in pink.

Following the notation of [2], we build a total of 30 hydrogen-bonded nucleic acid base pairs as shown in Scheme 1. For each complex, the nucleic acid bases are denoted by one or two letter abbreviations, among which are the four DNA bases: adenine (A), cytosine (C), guanine (G), and thymine (T), as well as fluorouracil (FU), hypoxanthine (HX), and uracil (U). For the most common configurations, we use the usual abbreviations: Watson–Crick (WC), reversed Watson–Crick (RWC), Hoogsteen (H), and reversed Hoogsteen (RH). We also use numbers to distinguish between different configurations of the same complex and not to introduce any kind of ordering. 

#### 4.3.2. Amino Acids, Dipeptides, and Tripeptides

**Scheme 2:**
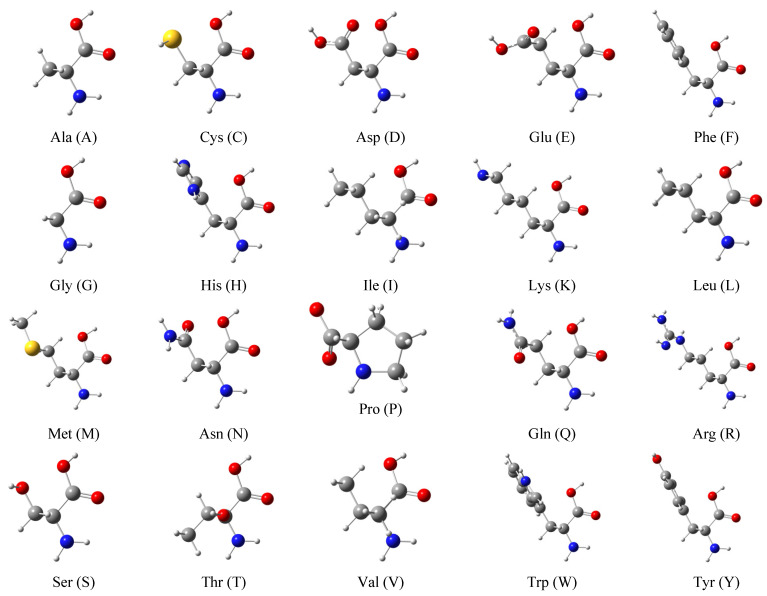
Schematic representation of 20 amino acids that form the basis of 400 dipeptides and 8000 tripeptides. Each molecule is named with a three-letter abbreviation and single-letter in the parenthesis. Color code: H in white, C in grey, N in blue, O in red, and S in yellow.

Shown in Scheme 2 are the 20 natural amino acids, directly taken from the template of the GaussView [58] program. We used the tleap module in the AmberTools package to generate a series of capped (ACE and NME) dipeptides (400) and tripeptides (8000). For each of the peptides, a simulation box was built with water molecules and Na^+^ and Cl^−^ ions. We performed a total of 1 ns MD simulations with the ff19SB [59] force field for the peptide and TIP3P [60] for the water molecules. An NPT (1 atm, 300 K) ensemble was used with a time step of 2 fs. The temperature was controlled with underdamped Langevin simulations of the “virtual” solvent with the damping coefficient *γ* = 5 ps^−1^ [61]. The pressure was held constant by applying the Langevin piston method [62,63]. Periodic long-range electrostatic interactions were computed using the particle mesh Ewald summation [64]. Covalent bonds associated with hydrogen atoms were constrained by the SHAKE algorithm [65]. All classical MD simulations were carried out with the Amber20 [66,67] CUDA version. A total of 10 MD snapshot structures of each peptide were evenly extracted for further quantum chemical calculations. Since we were not going to locate the global minimum conformers, we first performed single-point calculations of the 10 structures at the semi-empirical PM7 [68] level, and selected the structure with the lowest energy for subsequent analysis. 

All density functional theory (DFT) calculations were conducted with the Gaussian 16 [69] package with ultrafine integration grids and tight self-consistent field convergence. 

A full geometric optimization at the M06-2X/Def2-SVP level without any symmetry constraint was conducted for 30 base pairs and 8420 peptides. The optimized Cartesian coordinates are supplied in the Supplementary Materials. Since it is computationally demanding to perform harmonic vibrational frequency analysis for all systems in this study, we chose 30 base pairs, 20 amino acids, 400 dipeptides, and 10 randomly selected tripeptides and no imaginary frequencies were observed. Molecular polarizabilities, volumes, and wavefunctions were obtained at the M06-2X/Def2-TZVP level. Note that only the isotropic molecular polarizability α_iso_ = (α_xx_ + α_yy_ + α_zz_)/3 is reported in this work. Total energy components were obtained via the keyword iop(5/33 = 1). The Multiwfn 3.8 [70] program was utilized to calculate all ITA quantities introduced above by using the checkpoint or wavefunction file from the Gaussian calculations as the input. Molecular volumes were obtained at 0.001 e/Bohr*^3^* contour surface of electronic density. The stockholder Hirshfeld partition scheme of atoms in molecules was employed when atomic contributions were concerned. The reference density was the neutral atom calculated at the same level of theory as molecules.

## 5. Conclusions

In this work, we have explored the interaction energy of base pairs and molecular properties of both base pairs and amino acids, dipeptides, and tripeptides. Using the total energy decomposition schemes, one can find that the exchange-correlation effect makes the predominant contribution to the molecular stability, followed by the electrostatic potential, and steric hindrance plays a trivial role. We further revealed that molecular polarizabilities can be linearly correlated with ITA quantities. These strong linear correlations can be used to predict the molecular polarizabilities of larger peptides and even proteins. We mention in passing that two directions merit further studies. (i) Verification of the wide applicability of ITA quantities to various systems needs intensive work. (ii) When a fragment-based method, such as the linear-scaling generalized energy-based fragmentation (GEBF) approach, is taken into consideration, one can directly predict the subsystem (fragment of a large system, usually with a few atoms within a distance threshold) polarizabilities rather than solving the derivatives of molecular orbitals and obtain the molecular polarizability of macromolecular systems via a linear combination of subsystem polarizabilities. More work along this line is ongoing, and results will be presented elsewhere in the very near future.

## Figures and Tables

**Table 1:** Benchmark results of isotropic polarizabilities (*α*_iso_) of several density functionals and basis sets for 20 amino acids. ^a^ Units are in Bohr*^3^*.

Methods	6-311G(d,p)		Def2-SVP		Def2-TZVP		aug-cc-pVTZ	
	MUE ^c^	MSE ^d^	MUE	MSE	MUE	MSE	MUE	MSE
M06-2X	−15.3	15.3	−18.4	18.4	−8.9	8.9	−3.7	3.8
B3LYP	−10.7	10.7	−13.6	13.6	−4.6	4.6	−0.3	1.6
CAM-B3LYP	−13.9	13.9	−1.3	2.2	−7.8	7.8	−4.6	4.6
PBE0	−12.4	12.4	−15.2	15.2	−6.0	6.0	−1.9	2.6
ωB97XD	−14.2	14.2	−17.1	17.1	−8.1	8.1	−5.1	5.2
MP2 ^b^	−15.6	15.6						

^a^ The experimental data are taken from [32]; ^b^ MP2 results are taken from [33]; ^c^ **MUE**: mean unsigned error; ^d^ **MSE**: mean absolute error.

**Table 2:** Total energy difference (ΔE) and its components obtained at the M06-2X/Def2-TZVP level for 30 base pairs, including kinetic (ΔT_s_), exchange-correlation (ΔE_xc_), electrostatic potential (ΔE_e_), steric hindrance (ΔE_s_), and quantum effect (ΔE_q_). Units are in kcal/mol.

Base Pair	ΔT_s_	ΔE_x_	ΔE_c_	ΔE_xc_	ΔE_e_	ΔE_s_	ΔE_q_	ΔE
C-G-WC	12.1	−1.5	−16.6	−18.0	−21.4	−392.3	386.4	−27.3
G-G-1	8.2	−2.4	−17.4	−19.7	−14.6	−421.0	409.4	−26.1
C-HX	11.3	−0.4	−13.7	−14.1	−18.4	−300.3	297.5	−21.1
T-G-3	7.8	−1.6	−13.2	−14.8	−14.0	−325.5	318.5	−21.0
G-G-3	6.3	−0.9	−12.5	−13.5	−11.5	−310.0	302.9	−18.6
C-C-1	13.1	0.3	−13.0	−12.7	−20.2	−268.2	268.6	−19.9
T-G-1	4.5	−0.8	−12.3	−13.1	−7.7	−298.9	290.4	−16.2
A-G-1	9.3	1.2	−13.5	−12.3	−13.3	−273.0	270.0	−16.3
T-G-2	4.7	−0.6	−11.8	−12.4	−8.0	−286.7	279.1	−15.7
C-A-1	10.1	0.8	−11.3	−10.5	−14.5	−235.8	235.4	−14.9
T-A-H	6.3	0.8	−12.0	−11.2	−9.6	−270.3	265.4	−14.5
T-A-RH	6.2	0.8	−12.0	−11.2	−9.4	−270.0	264.9	−14.4
C-G-1	5.6	0.2	−11.4	−11.2	−8.8	−259.9	254.2	−14.5
C-A-2	7.8	1.5	−11.3	−9.8	−12.4	−232.2	230.2	−14.4
FU-A	6.7	0.6	−12.7	−12.1	−9.1	−277.6	272.2	−14.5
T-A-WC	6.7	0.8	−12.1	−11.3	−9.9	−270.7	266.0	−14.5
U-A	6.6	0.7	−12.5	−11.8	−8.9	−272.9	267.6	−14.1
A-G-3	8.2	2.0	−12.6	−10.6	−12.9	−249.8	247.5	−15.3
T-A-RWC	6.1	0.8	−12.0	−11.2	−9.4	−269.9	264.9	−14.4
A-A-1	8.7	0.9	−10.2	−9.3	−11.3	−215.8	215.1	−11.9
A-G-4	1.9	1.5	−10.1	−8.6	−3.7	−224.8	218.1	−10.4
C-T-2	6.5	1.3	−11.9	−10.6	−8.4	−245.6	241.6	−12.4
A-A-2	5.7	1.4	−9.3	−8.0	−8.7	−202.1	199.8	−10.9
T-T-1	3.6	0.1	−9.7	−9.5	−5.8	−226.1	220.3	−11.7
T-T-2	4.6	0.0	−9.8	−9.8	−6.7	−229.4	224.1	−11.9
T-T-3	3.8	0.2	−9.5	−9.3	−6.1	−223.2	217.7	−11.6
C-T-1	6.1	1.5	−11.7	−10.2	−8.0	−239.4	235.3	−12.0
A-G-2	1.9	1.5	−10.1	−8.6	−3.7	−224.4	217.8	−10.4
A-A-3	5.3	1.4	−9.3	−7.9	−8.3	−201.8	199.2	−10.9
G-G-4	−0.9	0.8	−11.0	−10.1	1.1	−256.2	245.2	−9.9

**Table 3:** Molecular polarizabilities (in Bohr*^3^*), molecular volumes (in Bohr*^3^*/mol), and ITA quantities (in a.u.) for 30 base pairs.

Base Pair	Polar	Vol	S_S_	I_F_	S_GBP_	E_2_	E_3_	^r^R_2_	^r^R_3_	G_1_	G_2_	G_3_
C-G-WC	170.2	1984.1	99.2	5854.3	924.3	866.2	48,121.2	138.8	143.9	−22.5	9.3	169.0
G-G-1	197.2	1954.2	109.9	6775.8	1060.1	1002.5	55,241.1	159.1	164.8	−23.4	8.7	194.8
C-HX	159.1	1863.3	93.6	5514.6	870.1	814.0	45,149.3	130.6	135.6	−23.6	11.2	157.8
T-G-3	180.1	2060.0	105.0	6215.6	978.7	927.0	53,287.8	147.0	152.7	−26.6	12.1	177.2
G-G-3	194.4	2184.6	110.2	6777.3	1060.3	1002.5	55,250.6	159.1	164.9	−23.6	8.7	194.0
C-C-1	144.9	1731.0	88.8	4934.0	788.7	730.0	41,006.7	118.5	123.3	−21.5	9.9	142.1
T-G-1	177.3	2058.5	105.1	6216.0	978.7	926.9	53,282.0	147.0	152.7	−26.7	12.5	177.1
A-G-1	191.7	2146.7	108.8	6327.7	1005.6	921.3	48,236.1	151.0	156.6	−24.7	10.6	186.1
T-G-2	177.2	2016.1	105.1	6216.1	978.8	926.9	53,283.8	147.0	152.7	−26.7	12.3	177.0
C-A-1	165.7	1707.8	98.2	5406.3	869.9	785.1	41,120.1	130.7	135.9	−23.5	10.5	159.8
T-A-H	169.8	2060.1	103.7	5766.3	924.1	845.6	46,267.5	138.9	144.5	−27.5	13.9	169.1
T-A-RH	169.6	1997.3	103.8	5766.3	924.1	845.7	46,272.0	138.9	144.5	−27.5	13.8	169.0
C-G-1	147.8	1899.5	99.6	5856.5	924.6	866.3	48,137.5	138.8	144.0	−22.8	9.7	167.6
C-A-2	164.4	1810.0	98.2	5406.4	869.9	785.1	41,118.5	130.7	135.9	−23.6	10.8	159.9
FU-A	158.2	1839.0	90.1	6089.2	925.1	933.8	59,941.1	138.6	143.4	−23.2	10.5	162.2
T-A-WC	169.7	1950.4	103.7	5766.3	924.1	845.6	46,264.0	138.9	144.5	−27.5	14.0	169.1
U-A	157.9	1892.1	93.6	5514.7	870.1	814.0	45,157.7	130.6	135.6	−23.7	11.0	157.6
A-G-3	190.2	2205.6	108.9	6328.0	1005.6	921.4	48,248.3	151.0	156.6	−24.8	10.1	186.1
T-A-RWC	169.7	2093.3	103.8	5766.3	924.1	845.7	46,279.4	138.9	144.5	−27.5	13.4	169.0
A-A-1	186.9	2020.8	107.6	5878.4	951.0	840.0	41,215.1	142.9	148.5	−25.5	12.0	177.5
A-G-4	189.6	2119.7	109.0	6328.3	1005.7	921.3	48,241.8	151.0	156.6	−24.7	10.4	185.3
C-T-2	150.7	1701.9	94.4	5294.8	843.0	790.8	46,189.2	126.7	131.9	−25.7	12.1	150.9
A-A-2	185.3	2157.6	107.7	5878.6	951.0	840.0	41,219.9	142.9	148.5	−25.6	11.8	177.6
T-T-1	158.4	1822.9	100.1	5655.5	897.3	851.3	51,315.6	135.0	140.6	−29.8	16.3	159.7
T-T-2	158.2	1918.8	100.1	5655.5	897.3	851.2	51,313.7	134.9	140.6	−29.8	16.5	159.7
T-T-3	158.7	1961.9	100.1	5655.5	897.3	851.3	51,318.3	135.0	140.6	−29.8	16.0	159.8
C-T-1	150.3	1874.4	94.4	5294.9	843.0	790.7	46,172.2	126.7	131.9	−25.7	12.9	150.8
A-G-2	189.6	2023.7	109.0	6328.3	1005.7	921.2	48,235.2	151.0	156.6	−24.7	10.7	185.4
A-A-3	185.3	2114.5	107.7	5878.6	951.0	840.0	41,217.5	142.9	148.5	−25.6	11.9	177.4
G-G-4	195.4	2115.8	110.3	6777.9	1060.4	1002.5	55,256.4	159.0	164.7	−23.7	9.2	193.4
R^2^	1.000	0.632	0.834	0.722	0.824	0.544	0.048	0.826	0.828	0.007	0.141	0.904

**Table 4:** Comparison of molecular polarizabilities (*α*_iso_) of 30 base pairs with conventional data as reference. Units of *α*_iso_ are in Bohr^3^ and G_3_ is in a.u.

Base Pair	*α*_iso_	Other Work ^a^			This Work
		Becke	Hirshfeld	avg.	G_3_
C-G-WC	170.2	128.7	188.1	158.4	169.0
G-G-1	197.2	144.9	213.1	179.0	194.8
C-HX	159.1	122.0	177.4	149.7	157.8
T-G-3	180.1	135.3	198.3	166.8	177.2
G-G-3	194.4	145.2	212.6	178.9	194.0
C-C-1	144.9	112.9	163.1	138.0	142.1
T-G-1	177.3	135.5	198.2	166.9	177.1
A-G-1	191.7	142.3	207.9	175.1	186.1
T-G-2	177.2	135.5	198.3	166.9	177.0
C-A-1	165.7	126.2	182.9	154.5	159.8
T-A-H	169.8	132.3	193.4	162.8	169.1
T-A-RH	169.6	132.3	193.4	162.9	169.0
C-G-1	169.9	129.2	187.5	158.4	167.7
C-A-2	164.4	126.3	183.2	154.7	159.9
FU-A	158.2	122.1	177.8	150.0	162.2
T-A-WC	169.7	132.3	193.4	162.8	169.1
U-A	157.9	122.0	177.4	149.7	157.6
A-G-3	190.2	142.4	208.0	175.2	186.1
T-A-RWC	169.7	132.3	193.4	162.9	169.0
A-A-1	186.9	139.4	202.6	171.0	177.5
A-G-4	189.6	142.5	207.5	175.0	185.3
C-T-2	150.7	119.3	173.4	146.4	150.9
A-A-2	185.3	139.5	202.8	171.2	177.6
T-T-1	158.4	125.8	183.6	154.7	159.7
T-T-2	158.2	125.8	183.6	154.7	159.7
T-T-3	158.7	125.8	183.7	154.7	159.8
C-T-1	150.3	119.4	173.4	146.4	150.8
A-G-2	189.6	142.5	207.5	175.0	185.4
A-A-3	185.3	139.5	202.8	171.2	177.4
G-G-4	195.4	145.5	212.1	178.8	193.4
MUE(%) ^b^		−23.4	11.6	−5.9	−1.2
MSE(%) ^c^		23.4	11.6	5.9	1.5

^a^ Tkatchenko–Scheffler approach on top of Becke or Hirshfeld partitions. ^b^ **MUE**: mean unsigned error. ^c^ **MSE**: mean signed error.

## Data Availability

Data is contained within the article.

## Supplementary Materials

The following supporting information can be downloaded at: https://www.mdpi.com/article/10.3390/ph15080938/s1, The optimized Cartesian coordinates of 30 base pairs and 20 amino acids, 400 peptides, and 8000 tripeptides.
